# In Situ Analysis of Li Plating and Stripping Behaviors Under Dynamic Current Conditions for Realistic Application Scenarios

**DOI:** 10.1002/advs.202414396

**Published:** 2025-01-28

**Authors:** Yanpeng Guo, Xinqi Wei, Cheng Zeng, Xinyu Ji, Yao Liu, Shuhao Wang, Xizheng Liu, Tianyou Zhai, Huiqiao Li

**Affiliations:** ^1^ State Key Laboratory of Materials Processing and Die & Mould Technology School of Materials Science and Engineering Huazhong University of Science and Technology (HUST) Wuhan 430074 China; ^2^ Key Laboratory of Optoelectronic Chemical Materials and Devices (Ministry of Education) Jianghan University Wuhan 430056 China

**Keywords:** dynamic charging, In situ imaging, lithium deposition, lithium metal batteries

## Abstract

Lithium metal batteries are considered the holy grail for next‐generation high‐energy systems. However, lithium anode faces poor reversibility, unsatisfying cyclability and rate capability due to its uncontrollable plating/stripping behavior. While galvanostatic conditions are extensively studied, the behavior under more realistic application scenarios with variable inputs are less explored. Here, an in situ imaging platform using in‐plane microdevice configurations is developed to effectively investigate Li plating/stripping behavior under dynamic conditions. This platform offers high detectivity for analyzing the nuclei size, density, distribution, and growth location, rate, and mode. It is for the first time revealed that nuclei density and growth locations remain constant and are solely determined by the initial nucleation overpotentials during dynamic plating. A transition in growth modes from uniform granular growth to tip‐induced dendrite growth, and finally to directional growth among the dendrites is also observed. Guided by these findings, a dynamic plating protocol is proposed, which can greatly improve the Li reversibility and cycling stability. This work not only provides a novel approach to visualize the evolution of key nucleation and growth parameters, especially under variable inputs, but also offers valuable guidance for the future industrialization of metal batteries and the rational design of charging facilities.

## Graphical abstract

An in situ imaging platform with high detection capability with in‐plane configuration is proposed to visualize the nuclei size/density/distribution and growth location/rate/mode upon Li plating under realistic application scenarios with variable inputs. It is revealed for the first time that nuclei density and growth locations remain constant and are solely determined by the initial nucleation overpotentials during dynamic plating.

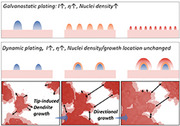

## 1 Introduction

Lithium metal batteries (LMB) have garnered significant attention over the past decade due to their exceptional energy density, positioning them as promising candidates for the future of the battery industry.^[^ ^1^, ^2^, ^3^, ^4^ ^]^ However, the direct use of metallic Li anodes presents challenges, including safety concerns and poor cycling performances, which impede the practical implementation of LMB.^[^ ^5^, ^6^, ^7^, ^8^ ^]^ These issues stem from the uncontrollable and complex plating and stripping behavior of metal anodes, which can lead to dendrite growth, dead Li formation, pulverization, and severe interfacial instabilities during cycling.^[^ ^9^, ^10^, ^11^, ^12^, ^13^ ^]^ It is well‐established that the applied current significantly influences the plating and stripping properties, particularly affecting the size, density, and distribution of Li nuclei, as well as growth patterns and dissolution modes, all of which are closely linked to the failure mechanisms of the Li metal anodes.^[^ ^14^, ^15^, ^16^, ^17^, ^18^ ^]^

Despite this, previous researches have primarily focused on the evolution of these nucleation, growth, and dissolution parameters under galvanostatic conditions, with little consideration given to dynamic current inputs.^[^ ^19^, ^20^, ^21^, ^22^, ^23^, ^24^ ^]^ In practical applications, charging and discharging conditions are often variable. For example, dynamic charging protocols are frequently used to reduce charging time.^[^ ^25^, ^26^, ^27^ ^]^ These protocols involve varying charging currents based on the state of charge (SOC), which leads to constant changes in overpotentials. In lithium‐ion batteries (LIBs), Li ions are extracted from the cathode and inserted into the crystal structure of the anode during charging.^[^ ^28^ ^]^ Variations in charging protocols do not alter the insertion/extraction behavior or the intrinsic number of active sites for lithium ions within the graphite anode. However, in LMBs, changes in applied currents could significantly affect nuclei density, size, distribution, growth rate, and locations, resulting in different growth and dissolution dynamics.^[^ ^15^, ^29^ ^]^ Another scenario is the pre‐activation process of LIBs, where extremely small currents are applied to maximize the activation of electrode sites and stabilize the electrode‐electrolyte interface.^[^ ^30^, ^31^, ^32^ ^]^ In LMBs, the morphology of deposits and extent of side reactions during activation are crucial for reversibility.^[^ ^33^ ^]^ Unfortunately, the evolution of nucleation and growth characteristics during Li plating/stripping with dynamic inputs has not been thoroughly explored due to the lack of suitable in situ characterization techniques and device configurations.

In this work, we propose using in situ optical imaging technique with a specially designed in‐plane device configuration to continuously monitor Li nucleation and growth under dynamically changing currents. In conventional face‐to‐face configurations, the overlapping morphologies of deposits at the interface make it difficult to inspect the evolution of nucleation and growth locations of individual Li clusters, especially with dynamic currents. In contrast, the in‐plane configuration offers high detectability for the distribution, size, and density of nuclei as well as the location, rate and patterns of Li growth and dissolution. The in situ imaging platform with high detection capability reveals, for the first time, that nuclei density and growth locations are determined solely by the initial Li plating process and remain constant even with subsequent dynamically changing currents. The transition from isotropic to directional growth modes of Li deposits is also observed with increasing currents, leading to closely‐connected deposition morphology. Guided by these in situ findings, we propose a dynamic plating protocol with high‐current nucleation and low‐current growth to enhance the reversibility and cycling stability of Li metal.

## 2 Results and Discussion

**Figure 1:**
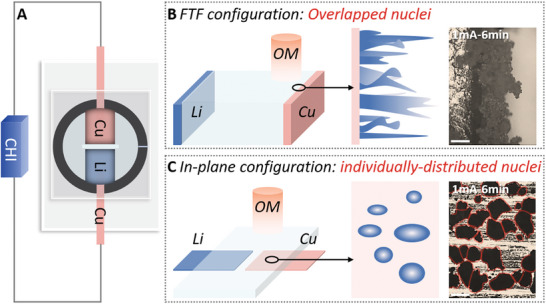
A) Schematic illustration of the in situ optical imaging platform. B) Face to face device configuration. C) In‐plane device configuration. Scale bar: 100 µm.

In situ optical imaging has been extensively used to assess the roughness of Li deposits on substrates, demonstrating the effectiveness of strategies aimed at achieving dendrite‐free Li plating (**Figure** **1**A).^[^ ^21^, ^34^ ^]^ Traditionally, a face to face (FTF) device configuration is employed, where the two electrodes are vertically aligned and the light path is perpendicular to the growth direction of Li deposits (Figure 1B). This configuration offers high detectability of growth rate and interfacial uniformity. However, due to the overlap of multiple Li clusters, it is challenging to monitor nucleation spots, cluster density, and growth locations on the whole substrate surface. To address these limitations, we developed an in‐plane configuration where the electrodes are collaterally placed, and the light path is parallel to the growth direction (Figure 1C). Additionally, the intensity distribution of the electric field can be used to study its impact on Li deposition and dissolution processes. Compared to the FTF configuration, the in‐plane device allows for high‐precision detection of nucleation sites, cluster density, and growth/dissolution locations, which are crucial for understanding the Li plating/stripping behavior.

**Figure 2:**
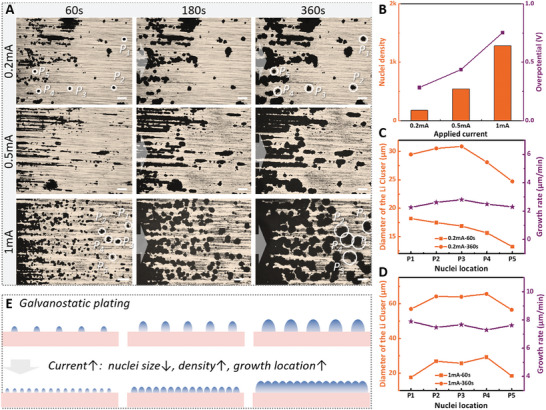
A) Real‐time optical images upon galvanostatic plating at 0.2, 0.5, and 1 mA using in‐plane devices. B) Nucleation overpotentials and nuclei density at varied currents using in‐plane device. Size evolution and growth rates of 5 selected Li clusters upon galvanostatic plating at C) 0.2 mA and D) 1 mA. E) Schematic illustration of Li plating characteristics under galvanostatic conditions. Scale bar: 100 µm.

The impact of current density on the nucleation and growth process was first investigated using this in‐plane configuration (**Figure** **2**A; Figures S1–S3, Supporting Information). Li foil and Cu foil were in‐plane placed on the glass slide with effective electrode area of 0.2 cm^2^ and an electrode channel of 2 mm. The injected electrolyte in all in situ test was the 1 m LiPF_6_ in EC: DMC: EMC (volume ratio, 1:1:1). Currents of 0.2, 0.5,, and 1 mA were applied to separate devices (electrode area: 0.2 cm^2^). The nucleation step completed within 60 s for all devices, with nuclei density increasing as the current increased. As plating continued, Li grew only near these primary nuclei rather than forming new clusters on the substrate, suggesting that adding a Li adatom to an existing nucleus is more energetically favorable. At 5 mA, the nucleation completed in less than 5 s, resulting in extremely high nuclei density, small size, and homogenous distribution (Figure S4, Supporting Information). However, high current also led to severe dendrite growth with prolonged plating. Figure 2B showed the nuclei density, counted by Image Pro Plus using optical images captured after 5 s of nucleation (Figure S5, Supporting Information), and nucleation overpotentials extracted from plating curves (Figure S6, Supporting Information). The nucleation phase is often characterized by a higher overpotential. The growth phase generally requires a lower overpotential as the energy barrier for adding atoms to existing nuclei is lower than forming new nuclei. As the current increased from 0.2 to 1 mA, the nucleation overpotential rose from 0.28 to 0.75 V, with nuclei density increasing from 178 to 1282 mm^−2^. This result aligned with classical theory and demonstrated the effectiveness of the in‐plane device for investigating nucleation characteristics during Li plating. The observed trends in voltage and nucleation overpotential were directly attributable to the applied current densities. The cell configuration and electrode distance could influence the absolute values of these potential parameters as results of additional ohmic polarization. Nonetheless, the effects of current density on plating morphological evolution remained consistent. Subsequently, the size evolution of five individual Li clusters at a specific current of 0.2 mA was quantitively analyzed (Figure 2C). Although the selected Li clusters, after 60 s of plating, were sparsely distributed over the substrate in distant regions with sizes ranging from 13.23 to 18.19 µm, their growth rates were similar, averaging 2.49 µm min^−1^. This indicated that the growth of Li deposits at low currents was less affected by the electric field distribution. Compared to the 0.2 mA condition, the sizes of Li clusters after 360 s of growth at 1 mA were much larger, averaging 61.5 µm. Accordingly, the growth rate at 1 mA was 7.59 µm min^−1^, much higher than that at 0.2 mA, suggesting that growth rate was largely determined by the applied current. Figure 2E provided a schematic illustration of Li plating/stripping characteristics under galvanostatic conditions based on our experimental results. Increasing current density could lead to increased nuclei density, reduced nuclei size, and faster growth. The growth location remained constant under a given current, manifesting only in enlarged Li cluster size and evolving growth patterns. During stripping, the shrinkage rates of Li deposits were 0.802 µm min^−1^ (0.2 mA) and 1.204 µm min^−1^ (1 mA). Notably, the growth rate increased ≈ 3‐fold as the current increased, whereas the shrinkage rate exhibited only a 1.5‐fold increase, indicating a more pronounced impact of current on the growth rate. At 1 mA, the shrinkage rate of Li deposit decreased by over sixfold compared to the growth rate. These distinctions reflected that the plating process followed a surface‐growth pattern with significant size increase, while the stripping process exhibited a core‐dissolution pattern, largely maintaining the outermost cluster shape (Figure S7, Supporting Information). The subsequent regeneration of Li deposits was also affected by the morphological properties post‐stripping (Figure S8, Supporting Information). After the first plating/stripping process, residual dead Li particles remained on the substrate. During subsequent plating, new nuclei formed on the exposed substrate rather than on the residual Li. Following Li growth also tended to occur near these newly formed Li nuclei. This behavior suggested that initial non‐uniformity could exacerbate the heterogeneity of the anode surface.

**Figure 3:**
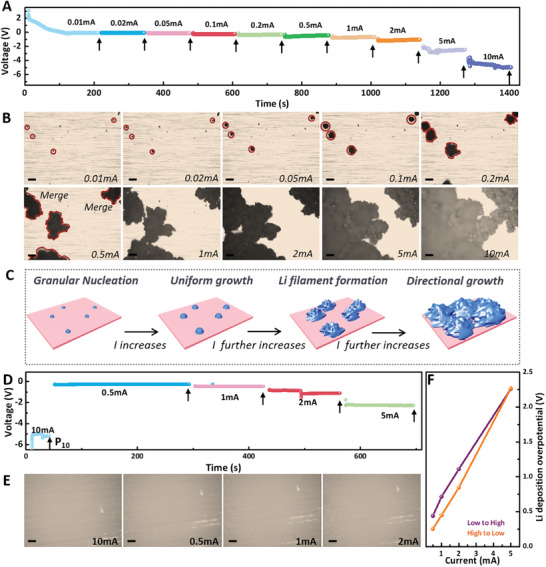
A) The electrochemical curve during the dynamic plating of Li metal on Cu substrate where the current was increased from 0.01 to 10 mA and B) corresponding in situ optical images at each terminal state as the arrow pointed. C) Schematic illustration of the nucleation and growth process of the dynamic plating. D) The electrochemical curve during the dynamic plating of Li metal on Cu substrate where the current was reduced from 10 to 0.5 mA and E) corresponding in situ optical images at each terminal state as the arrow pointed. F) A comparison of the deposition overpotentials of dynamic plating with current from high to low and from low to high. Scale bar: 100 µm.

This in situ imaging platform with an in‐plane configuration was applied for the first time to investigate the plating behavior of Li metal under more realistic scenarios owing to its high detectability of nuclei size, density, and locations. Typically, the galvanostatic charging was replaced by dynamic charging in practical applications to reduce the charging time. During dynamic charging, the applied currents changed dynamically with SOC values. The current was varied continuously from 0.01 to 10 mA, with each current applied for 2 min (**Figure** **3**A, electrode area: 0.2 cm^2^). The entire process was monitored in situ. According to the plating curve, the deposition plateau increased with the current, indicating an amplified driving force for ion transport. Figure 3B shows the optical images taken at each terminal state, as indicated by the arrows. After initial plating at an extremely low current of 0.01 mA, several granular Li embryos with dark color formed on the Cu substrate. When the current was switched to 0.02 mA, no new Li nuclei appeared. As the current increased to higher values, even up to 10 mA, the nuclei density and growth locations remained unchanged. The only variations in these Li clusters during dynamic plating were their changing growth rates at different currents and the gradually evolving growth patterns. The original granular Li nuclei underwent an initial step of uniform growth, followed by a second step of dendrite nucleation as the current progressively increases (Figure 3C). When the current increased further, a third step of directional growth between dendrite‐rich, irregular Li clusters was observed, due to the mismatch between ion concentration and electron distribution within the Li cluster. The Li plating process with current decreasing from high to low was also monitored in situ (Figure 3D,E). Initially, a current of 10 mA was applied to the cell, resulting in a large nucleation overpotential of 2.05 V and a highly homogenous distribution of numerous Li nuclei. When the current was successively reduced to lower values of 0.5, 1, and 2 mA, no new nuclei formed, and uniform Li growth was achieved. These observations demonstrated that nuclei density and growth location were solely dependent on the initial Li plating process, regardless of the subsequent applied currents and over‐potentials. Additionally, compared to nucleation at low current of 0.02 mA, nucleation at 10 mA at the initial stage contributes to decreased deposition overpotentials at the same currents (Figure 3F).

**Figure 4:**
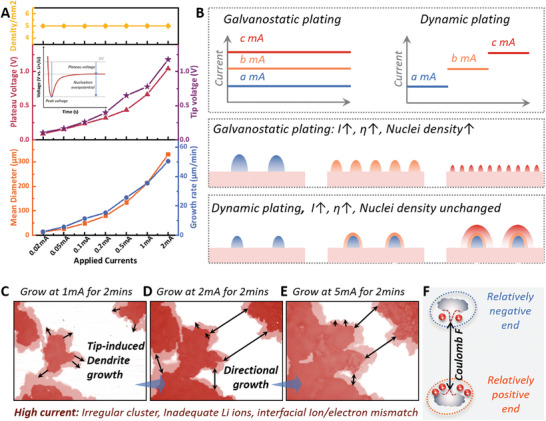
A) Evolution of the nuclei density, plateau and peak voltage, mean diameter, and growth rate of Li deposits during the dynamic plating. The nuclei density and growth locations remain unchanged though the nucleation overpotentials increase during dynamic plating. B) Schematic illustration of nucleation/growth modes under galvanostatic and dynamic plating. The growth of Li cluster at C) 1 mA for 2 min, D) 2 mA for 2 min, and E) 5 mA for 2 min. F) Schematic illustration of the mechanism for the directional growth. As the current increases, a directional growth among the tip‐induced dendrites was observed owing to the mismatch between interfacial ion and electron distribution, which resulted in the formation of relatively positive and negative ends. The consequential attractive coulomb forces could drive the directional growth.

A detailed quantitative analysis of the nucleation and growth properties during dynamic plating was conducted (**Figure** **4**A; Figures S9–S11, Supporting Information). The nuclei density and growth location remained constant at 5/mm^2^ as the current increased from 0.02 to 2 mA. The peak potential, which includes both nucleation and deposition overpotential, increased from 0.107 V at 0.02 mA to 1.177 V at 2 mA. Similarly, the plateau voltage for deposition rose from 0.090 V at 0.02 mA to 1.044 V at 2 mA, indicating intensified cell polarization for mass transport. The nucleation overpotentials were calculated to be 0.017 V at 0.02 mA and 0.133 V at 2 mA respectively. The mean diameter of the Li clusters during dynamic charging varied with current, increasing from 14.29 µm at 0.02 mA to 330.04 µm at 2 mA. Growth rates were calculated as 2.44 µm min^−1^ (0.02 mA), 5.73 µm min^−1^ (0.05 mA), 11.37 µm min^−1^ (0.1 mA), 15.39 µm min^−1^ (0.2 mA), 25.74 µm min^−1^ (0.5 mA), 35.52 µm min^−1^ (1 mA) and 50.41 µm min^−1^ (2 mA), showing a positive relationship with current. Figure 4B summarized the plating behavior of Li metal under the galvanostatic and dynamic plating conditions. In galvanostatic conditions, a constant current was applied throughout the plating process, aligning well with classic nucleation theory where increased nucleation overpotential results in new nuclei with higher density and smaller size. In dynamic plating, however, nuclei density and growth locations were determined solely by the initial applied current. Subsequent increases in current and nucleation overpotentials did not affect nuclei density and growth locations.

As the current progressively increased during dynamic plating, the growth pattern of Li metal evolved through three stages: a) uniform granular growth at low currents; b) the tip‐induced dendrite growth at moderate currents; c) directional growth among dendrites at high currents. The local ion concentration and electric field distribution are pivotal factors that influence the transitions in growth behavior during the dynamic plating process. Specifically, the local ion concentration is primarily governed by the ion transport kinetics within the electrolyte and the distribution of in‐built electric field. The electric field distribution is significantly influenced by the morphological properties of the deposits and the charge‐transfer dynamics. At low plating currents, Li deposits exhibited a relatively regular and isotropic morphology, facilitating homogeneous electron distribution at the frontend surface. Concurrently, the electrolyte supported efficient ion transport, ensuring smooth charge transfer, and ultimately leading to the uniform Li growth. At elevated currents that exceed the ion transport kinetics, the replenishment of ions cannot match their consumption during plating. As shown in Figure 4C and Figure S11 (Supporting Information), the growth rate varied at different locations within a single cluster during Li plating at 1 mA, with protrusions on the cluster surface with higher growth rates, suggesting the formation of dendrites. This was due to the positive feedback between dendrite growth and ion concentrations.^[^ ^35^ ^]^ Additionally, as the current increased further, dendrite growth demonstrated directionality (Figure 4D,E). Dendrites on one cluster tended to grow toward those on another cluster, eventually merging through a face‐to‐face (directional) growth mode. Dendrites growing in this mode exhibited higher growth rates compared to the tip‐induced mode. The mechanism of this directional growth was proposed in Figure 4F. At high currents, rapid ion depletion at the vicinity of Li cluster resulted in heterogeneous interfacial ion concentrations. The formation of dendrites with varying geometries also led to non‐unform electron distribution on the surface. Consequently, external Li ions around the anode cannot neutralize the internal electrons. This mismatch between ion and electron concentrations created relatively negatively and positively charged ends, resulting in attractive coulomb forces that drove directional growth among these dendrites. Simulations also demonstrated the pronounced localization of electric fields and significant concentration gradients particularly at the tips of the growing dendrites (Figure S12, Supporting Information). Guided by these findings, the prerequisite to avoid dendritic growth is to establish stable local charge neutrality at the anode‐electrolyte interface, which involves three key aspects: a) homogenization of electron distribution at the frontend surface of the plating electrode, necessitating uniform geometry and electronic properties of the Li deposits; b) leveling of ion concentration near the deposits, requiring rapid ion transport kinetics in the electrolyte; c) uniform charge‐transfer dynamics, involving compatible de‐solvation energy barriers and reaction activity of deposits.

**Figure 5:**
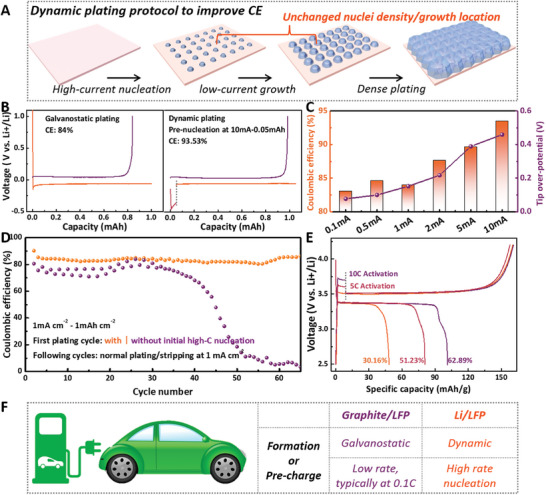
A) Schematic illustration of the dynamic plating protocol with an initial high‐rate nucleation and a following low‐rate growth. Plating/striping curves with galvanostatic plating and dynamic plating. The coulombic efficiency (CE) was improved from 84% to 93.53% via a facile dynamic plating. C) Peak voltages and coulombic efficiencies through dynamic plating with the initial nucleation current ranging from 0.1 to 10 mA (Fixed nucleation capacity of 0.05 mAh). As shown, higher nucleation rate leads to better Li reversibility. D) The coulombic efficiencies upon cycling with or without dynamic plating at the very first cycle. E) Charge‐discharge curves of anode‐free Cu‐LFP full cells with or without dynamic charging. F) A comparison of the formation and pre‐charge process of prevailing Li‐ion batteries and future Li metal batteries. In order to improve the Li reversibility, a dynamic charging with an initial high‐rate nucleation is highly suggested.

Inspired by the dynamic plating behavior, a strategy to enhance the reversibility of Li deposits was proposed, consisting of a two‐step process: high‐current nucleation followed by practical‐current growth (**Figure** **5**A). 2032‐type coin cells were assembled using Li foil and Cu foil with effective electrode area of 1.13 cm^2^. The electrolyte was the 1 m LiPF_6_ in EC: DMC: EMC (volume ratio, 1:1:1). Initially, nucleation was stimulated at a high current to create a uniform layer of Li nuclei. The current was then switched to a practical level (1 mA cm^−2^) for uniform Li growth. Figure 5B presented the galvanostatic Li plating/stripping curves at 1 mA cm^−2^ in an ester electrolyte, showing a low coulombic efficiency (CE) of 84%. A series of additional nucleation steps at currents ranging from 0.1 to 10 mA cm^−2^ were introduced with a fixed capacity of 0.05 mAh cm^−2^ before plating at 1 mA cm^−2^ (Figure S13, Supporting Information). The CE was increased to 93.53% with the introduction of 10 mA cm^−2^ nucleation (Figure 5C). Figure 5E summarized the CE values and peak voltages in response to the nucleation currents. Pre‐nucleation at currents below 1 mA cm^−2^ showed limited positive effects on CE. However, when the pre‐nucleation current exceeded 1 mA cm^−2^, CE increased with the nucleation currents. This improvement was attributed to the high nucleation overpotential at high currents, which promoted the formation of a highly uniform nuclei distribution with high density and small size. This nucleation characteristic facilitated homogenous Li growth on these primary nuclei. The total time taken to complete the stripping during dynamic charging can also be reduced by optimizing the nucleation current density and capacity (Figure S14, Supporting Information). Higher pre‐nucleation currents and capacities may also be applied, but the maximum workable current and capacity are primarily determined by the ion transport dynamics of different electrolyte systems. Additionally, a pre‐nucleation of 10 mA cm^−2^ and 0.05 mAh cm^−2^ at the first cycle of Li─Cu half cells significantly enhanced the cycling stability (Figure 5D). Without pre‐nucleation, strong fluctuations and a sharp drop in CE occurred due to poor Li reversibility. In contrast, dynamic plating resulted in higher and more stable CEs during cycling. The effect of dynamic plating with high‐rate nucleation on Li reversibility was also monitored in situ (Figures S15 and S16, Supporting Information). The CE of device using dynamic plating was 85.28%, ≈9% higher than that using galvanostatic plating. Moreover, the morphology of sparsely distributed Li clusters was transformed into a uniform Li layer through dynamic plating, enhancing the reversibility during subsequent stripping. Regarding rate capability, symmetric Li─Li cells initiated with 10 mA cm^−2^ demonstrated better stability, lower voltage hysteresis, reduced cell impedance, and smoother morphology compared to those started with 0.5 mA cm^−2^ (Figure S17, Supporting Information). This dynamic plating protocol was further applied to anode‐free Li metal batteries, where Cu and LiFePO_4_ served as the anode and cathode respectively. Cells with galvanostatic charging exhibited an extremely low CE of 30.16%. When dynamic charging, consisting of an initial high‐rate charging of 5% capacity followed by 1 C charging, was introduced, the CE increased to 51.23% (5 C‐nucleation) or 62.89% (10 C‐nucleation). Based on these results, special attention should be given to the formation and pre‐charge process of Li metal batteries during industrial fabrication (Figure 5D). For Li‐ion batteries, both the two processes were typically conducted at low rates (0.1 C) in galvanostatic mode to stabilize the SEI layer or maximize the nominal capacity. For Li metal batteries, a dynamic mode with initial high‐rate nucleation followed by low‐rate growth during the formation and pre‐charge process was highly recommended to improve the Li reversibility.

## 3 Conclusion

In this work, an in situ imaging platform with high detection capability was developed that integrated an optical microscope with a compatible monitoring window and in‐plane cell configurations. Its effectiveness in probing nuclei density and size, growth and dissolution locations and rates, as well as plating and stripping patterns, was quantitively verified. This platform was then used for the first time to investigate Li deposition behavior under realistic application scenarios with dynamic current inputs. It was revealed for the first time that nuclei density and growth locations remained unchanged during dynamic plating, determined solely by the initial plating current and overpotentials. Increases in both current and overpotentials resulted only in larger cluster sizes, accelerated growth rates, and transitions in growth modes from uniform granular growth, to tip‐induced dendrite growth and finally to directional growth among the dendrites. Guided by these findings, a dynamic plating protocol involving an initial high‐current nucleation followed by low‐rate growth was proposed, which significantly improved Li reversibility and cycling stability. The coulombic efficiency of an anode‐free Cu‐LFP full cell was increased by 32.73% using this dynamic charging tactic. This work effectively combined the advantages of the in‐plane configuration and in situ optical imaging, providing a powerful solution to visualize the evolution of key nucleation and growth parameters, especially under variable inputs. The results of this work offered valuable guidance for the future industrialization of Li metal batteries and the rational design of relevant charging facilities.

## Conflict of Interest

The authors declare no conflict of interest.

## Supporting information



Supporting Information

## Data Availability

The data that support the findings of this study are available in the supplementary material of this article.
