# Supplementary material for: In Situ Analysis of Li Plating and Stripping Behaviors Under Dynamic Current Conditions for Realistic Application Scenarios
Source: Adv Sci (Weinh). 2025 Jan 28;12(11):2414396. doi: 10.1002/advs.202414396 (PMC11923962; doi:10.1002/advs.202414396)
Supplement: Supplementary file 1 — Supporting Information [file ADVS-12-2414396-s001.docx]

Supporting Information

In-situ Analysis of Li Plating and Stripping Behaviors Under Dynamic Current Conditions for Realistic Application Scenarios

Yanpeng Guo^a^, Xinqi, Wei^a^, Cheng Zeng^a^, Xinyu Ji^a^,Yao Liu^a^, Shuhao Wang^b^, Xizheng, Liu^b^, Tianyou Zhai^a^, Huiqiao Li^a,b^*

Y. Guo, X. Wei, C. Zeng, Y. Liu, X. Ji, Prof. T. Zhai, Prof. H. Li

^a^State Key Laboratory of Materials Processing and Die & Mould Technology, School of Materials Science and Engineering, Huazhong University of Science and Technology (HUST), Wuhan 430074, China.

S. Wang, Prof. X. Liu

^b^Key Laboratory of Optoelectronic Chemical Materials and Devices (Ministry of Education), Jianghan University, Wuhan, 430056, China

E-mail: [hqli@hust.edu.cn](mailto:hqli@hust.edu.cn)

**Experimental Section**

**Set-up of the in-situ optical imaging platform**. The Cu foil and Li foil are vertically placed in the face to face (FTF) configuration while they are in-plane placed on the glass slide in the in-plane configuration. Thereinto, Li foil is adhered to the Cu foil via conductive silver paste to serve as the reference and counter electrode in the glove box. The two electrodes are sealed by a fluororubber O-ring and another cover glass using the organic-resistant hot sealing glue. Two slits are made on the O-ring for electrolyte injection. The electrolyte used in all in-situ test is the 1 M LiPF_6_ in EC: DMC: EMC (volume ratio, 1:1:1). After the electrolyte injection, the two slits are sealed by the glue before testing outside the glovebox. The optical microscope (OLYMPUS) is focused on the Cu-electrolyte interface in the FTF configuration while that is focused on the top of Cu in the in-plane configuration. For in-situ imaging, the two electrodes are connected to an external electrochemical workstation (Biologic SP-150) in which the Cu is the working electrode and Li is the counter/reference electrode.

**Electrochemical Measurements**. All cells were assembled using CR2032 type coin cells in glovebox and tested on LAND battery testing system. The electrolyte was 1 M LiPF_6_ in EC: DMC: EMC (volume ratio, 1:1:1). For the coulombic efficiency test, Cu-Li asymmetric cells were assembled (Cu: diameter 14 mm, thickness 9 μm; Li: diameter 12.5 mm, thickness 0.55 mm). An initial plating at high currents and a fixed capacity of 0.05 mAh is introduced to investigate the impact of nucleation status on the Li reversibility. Cu-LFP anode-free cells were also assembled where the Cu and LFP serve as the anode and cathode respectively (LFP: diameter 10 mm). To prepare the LFP cathode, the carbon-coated LFP powder was mixed with conductive agent C45 and PVDF binder (PVDF in NMP: 40 mg/ml) according to a mass ratio of 8:1:1. Then the slurry is sealed in a 5 ml bottle and vigorously stirred for 2h for further casting onto the aluminum foil. The thickness during slurry casting was set at 250 μm and the drying condition was 80 °C for 12 h.

**
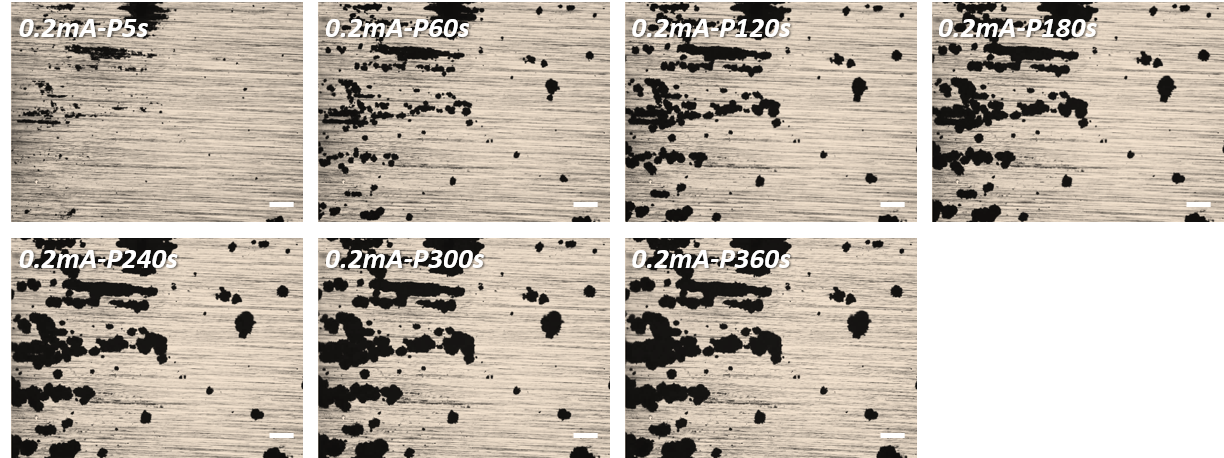
**

**Figure S1**. In-situ optical images upon galvanostatic plating at 0.2 mA. Scale bar: 100 μm.


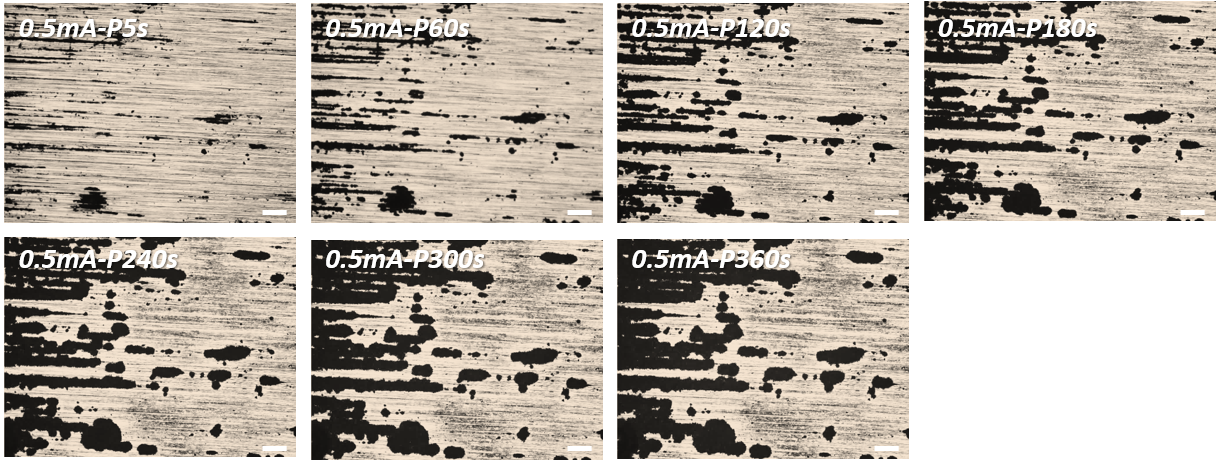


**Figure S2**. In-situ optical images upon galvanostatic plating at 0.5 mA. Scale bar: 100 μm.


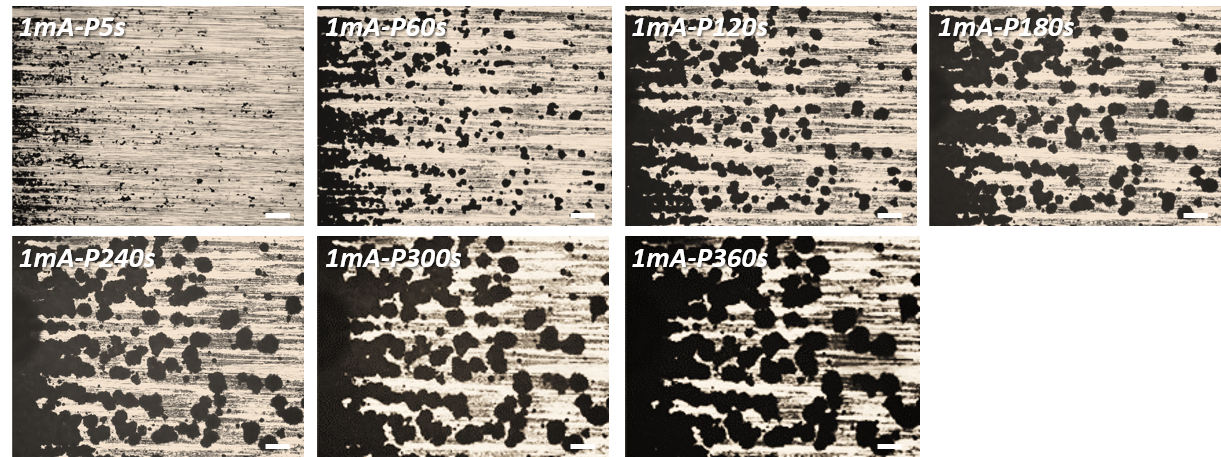


**Figure S3**. In-situ optical images upon galvanostatic plating at 1 mA. Scale bar: 100 μm.


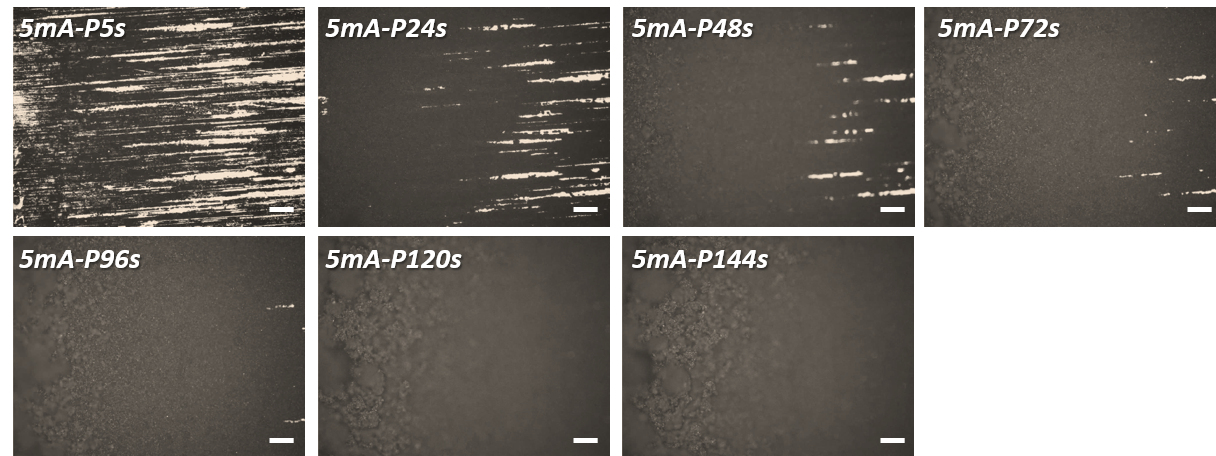


**Figure S4**. In-situ optical images upon galvanostatic plating at 1 mA. Scale bar: 100 μm.


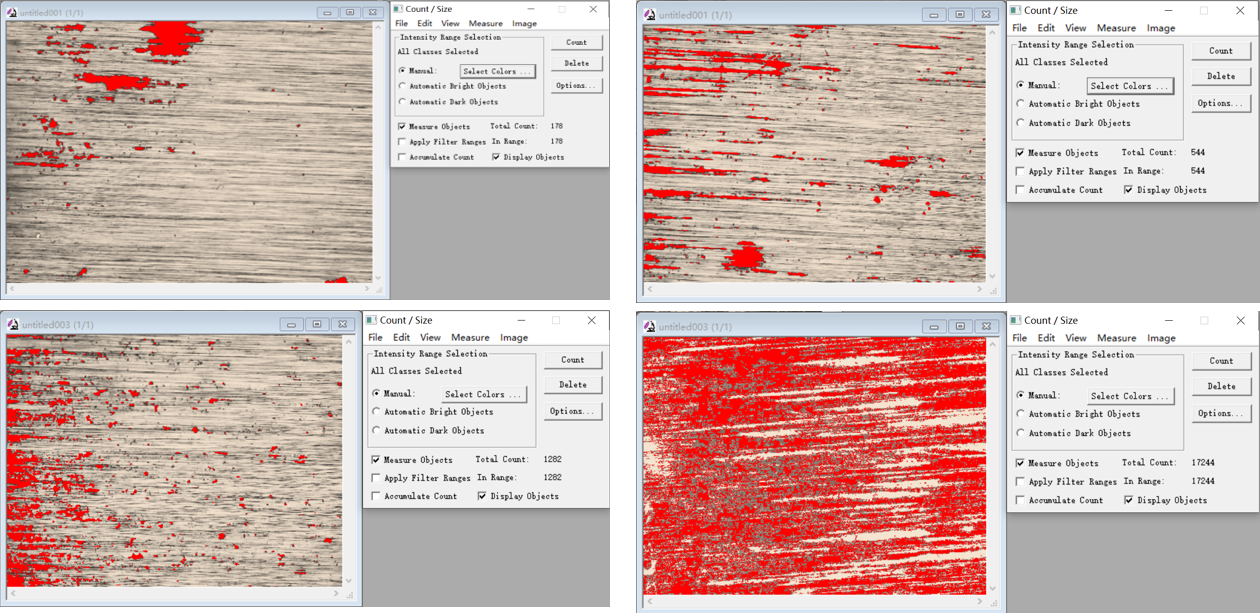


**Figure S5**. Nuclei density counted by the Image Plus Pro. The optical images are captured after plating for 5 seconds at varied currents.


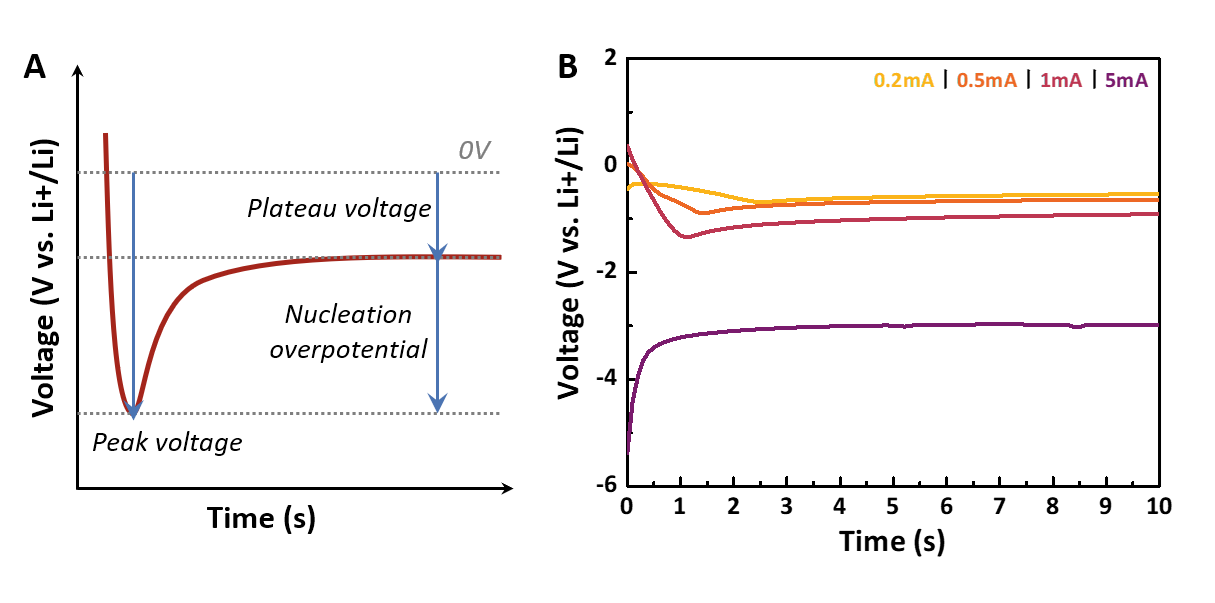


**Figure S6**. a) Schematic illustration of the peak voltage, plateau voltage and nucleation overpotential. b) Plating curves of in-plane devices during initial nucleation at varied currents.


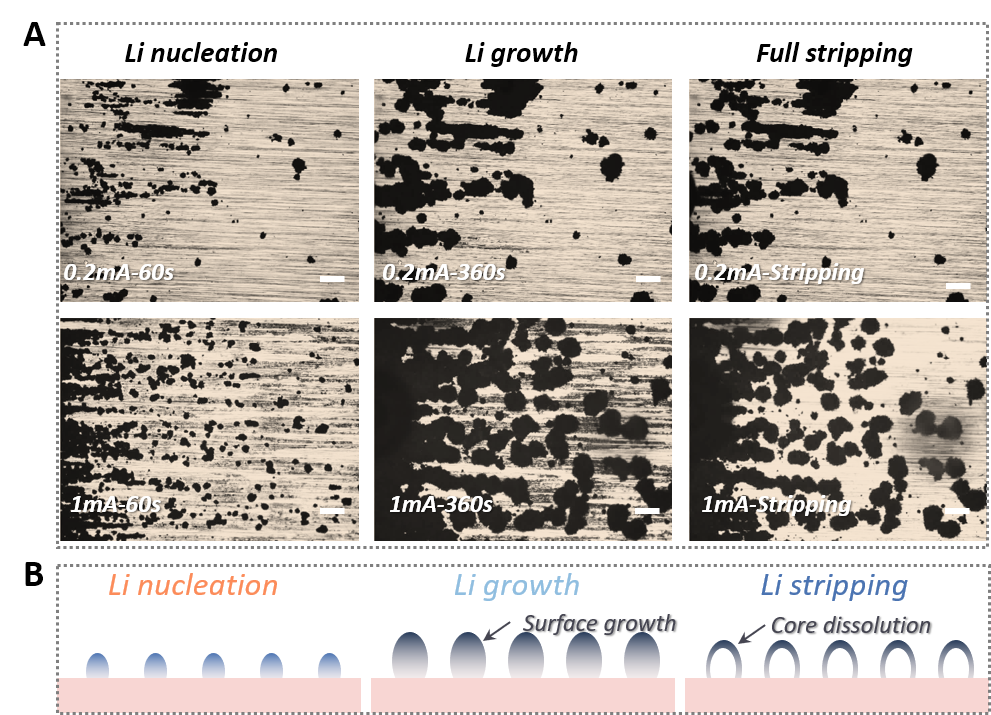


**Figure S7**. a) Optical images of Li deposits after nucleation, growth, and full stripping at two different currents. b) Schematic illustration of potential growth and stripping mechanisms which involved a surface growth during plating and a core dissolution upon stripping.


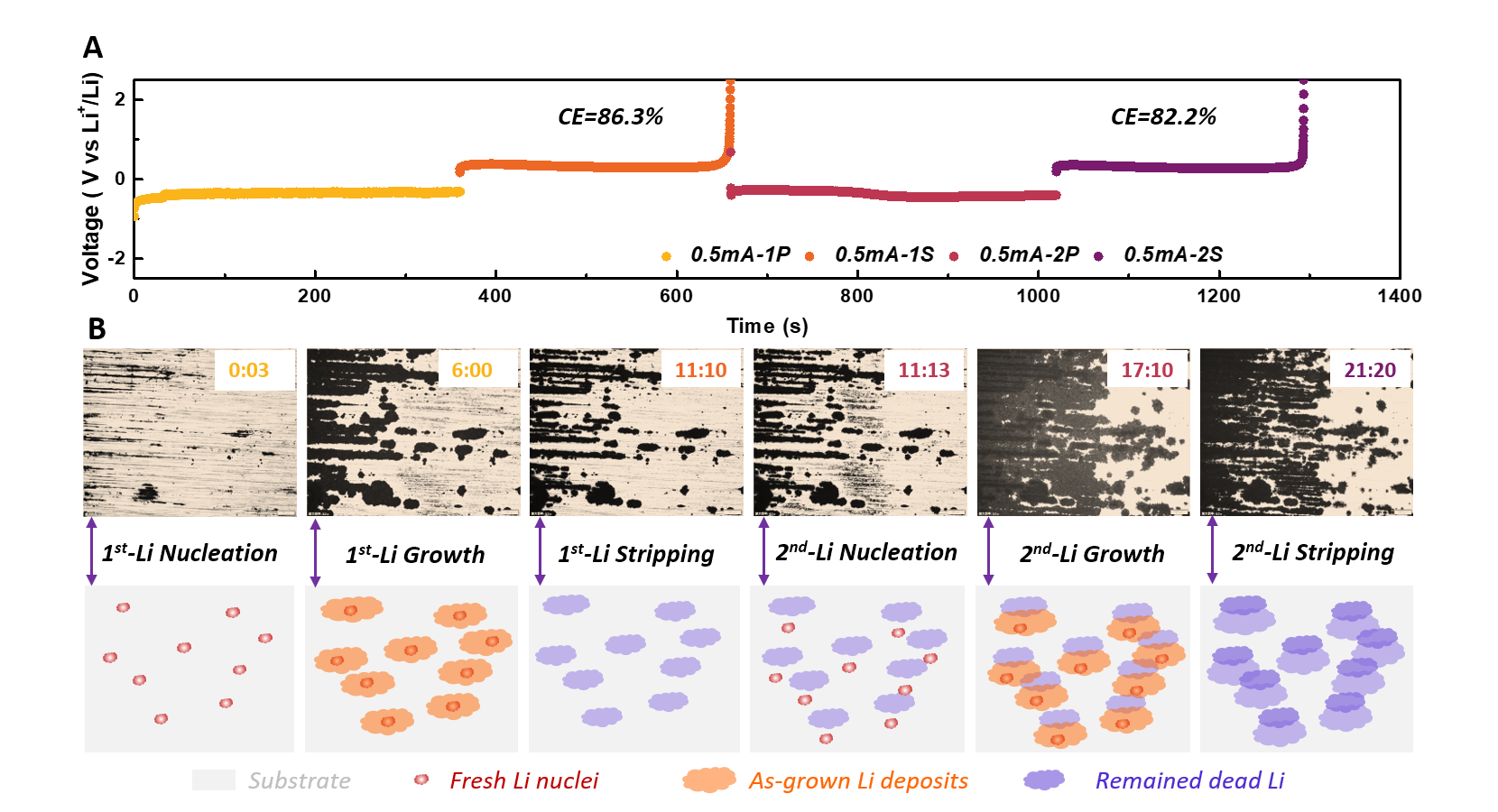


**Figure S8**. a) Voltage profiles of two plating/stripping cycles at a current of 0.5 mA. b) Corresponding in-situ optical images and schematic illustration of Li deposits after nucleation, growth, and full stripping at the 1^st^ and 2^nd^ cycle.


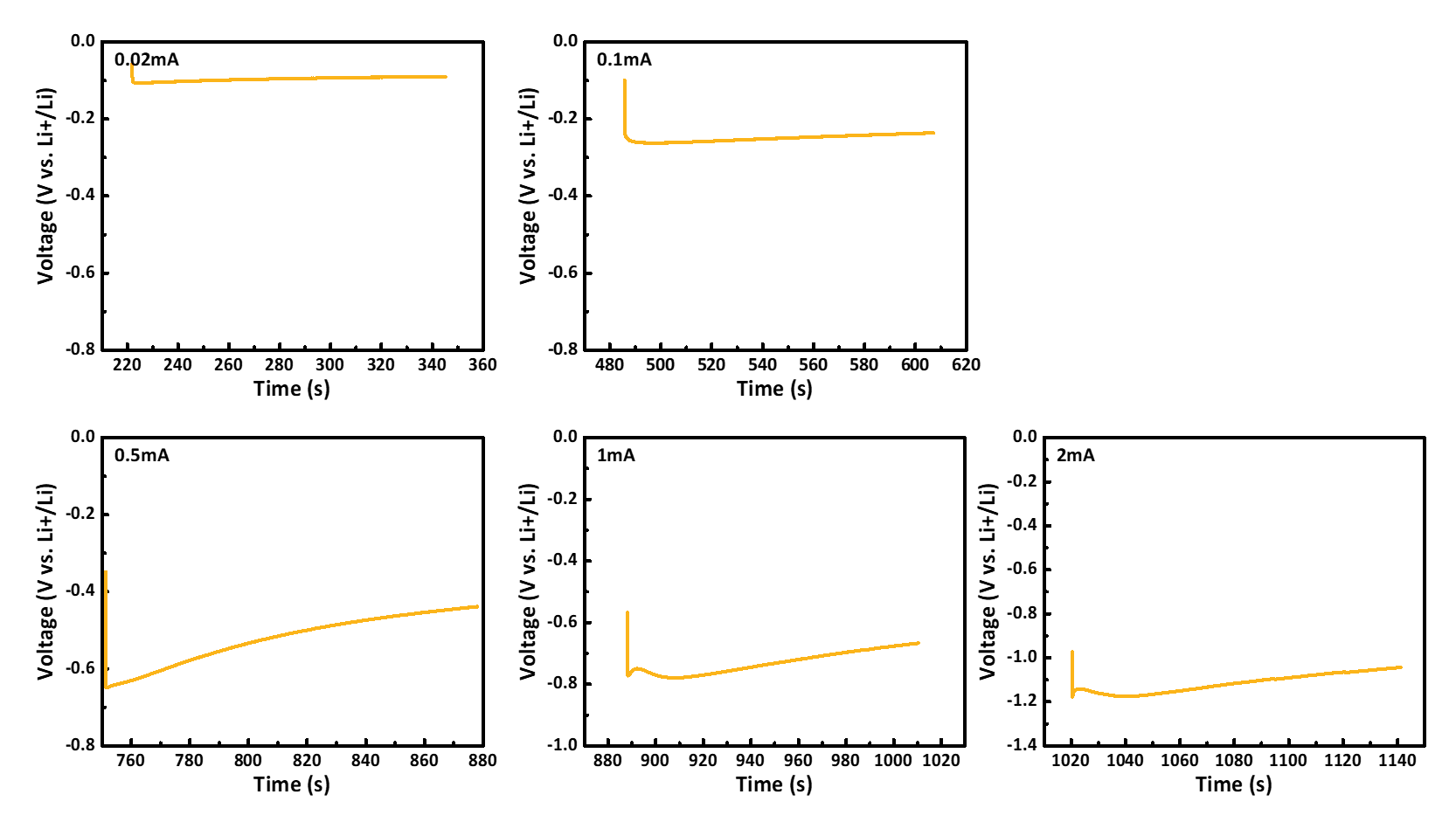


**Figure S9**. Voltage-time profiles at varied currents during the step-wise plating.


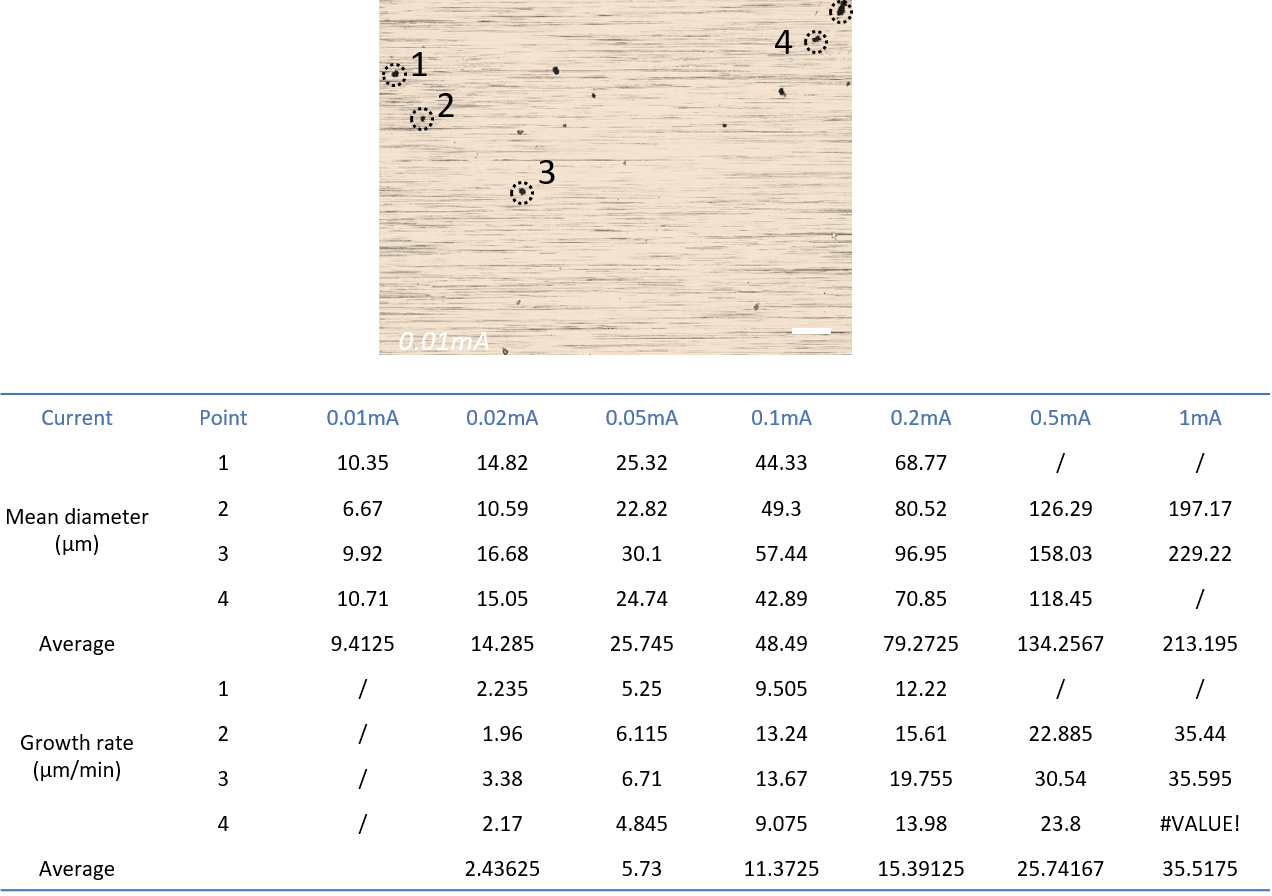


**Figure S10**. Size evolutions of Li clusters during step-wise charging and calculated growth rates at varied currents. scale bar: 100 μm.


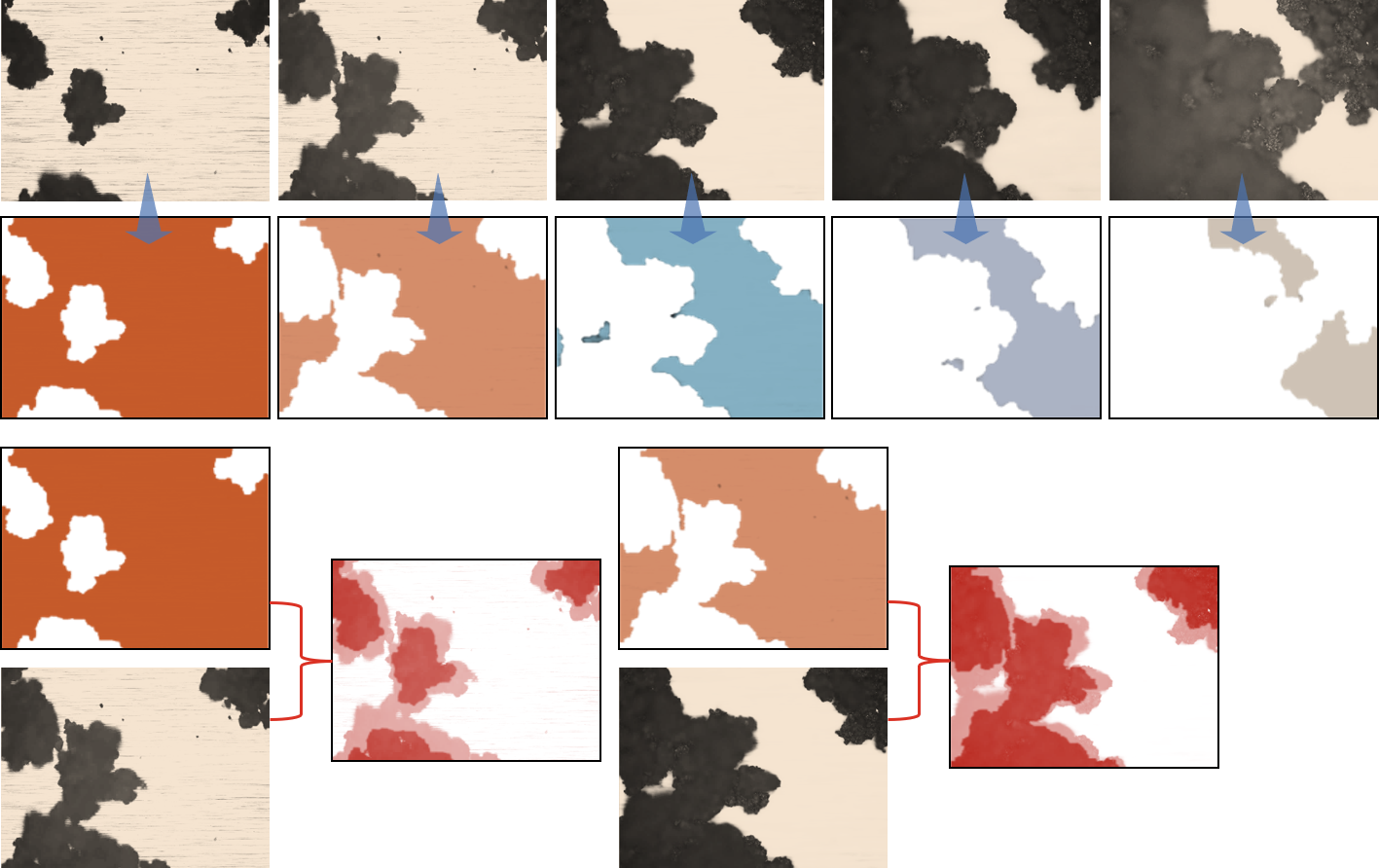


**Figure S11**. Figure treatment methods to show the transition of growth mode during step-wise plating.


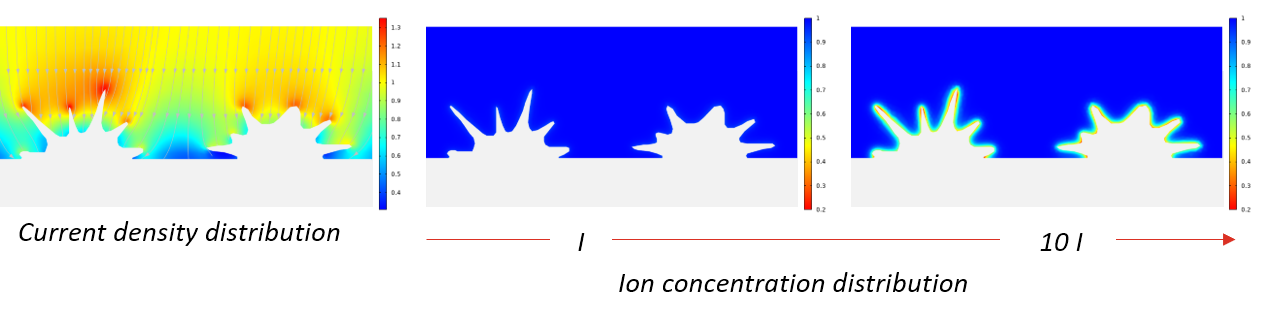


**Figure S12**. Simplified simulations of current density distribution at the dendrite surface and the ion concentration gradients in the electrolyte at bare current and a tenfold increased current.


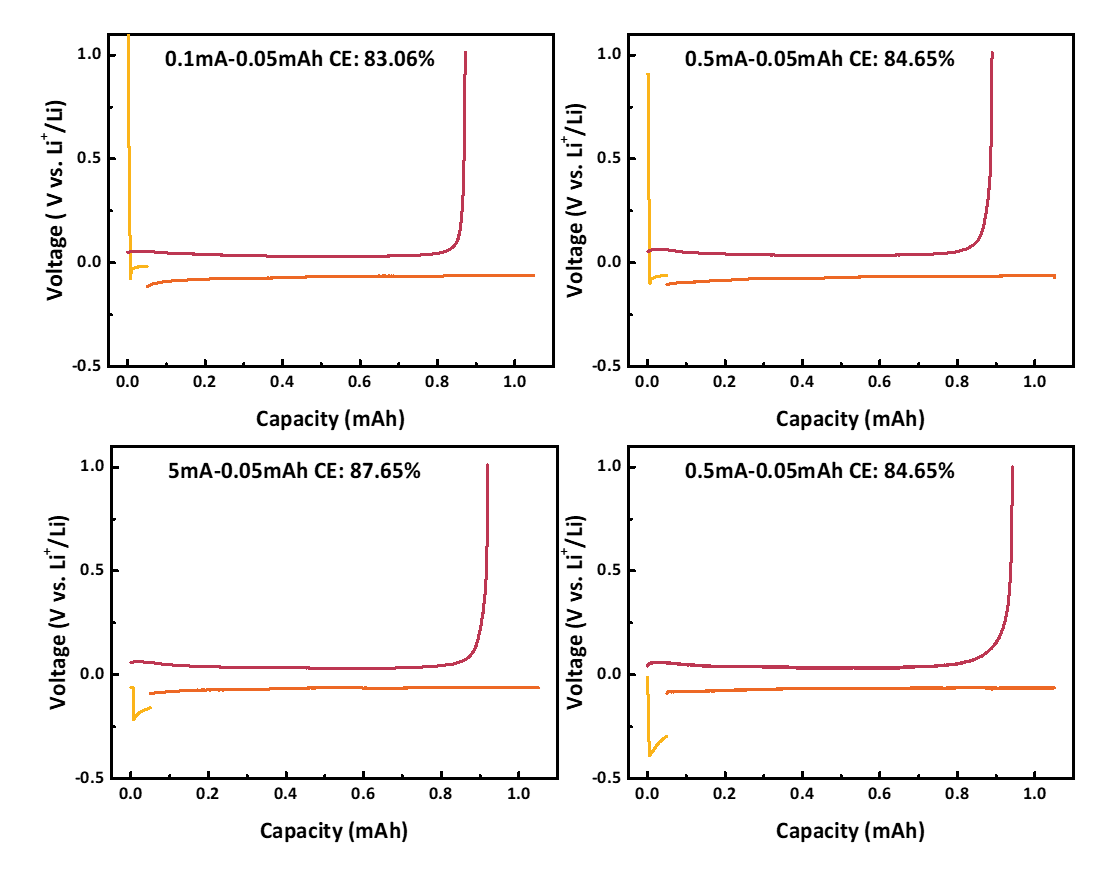


**Figure S13**. Li plating/stripping curves with a 0.05mAh nucleation at varied currents.


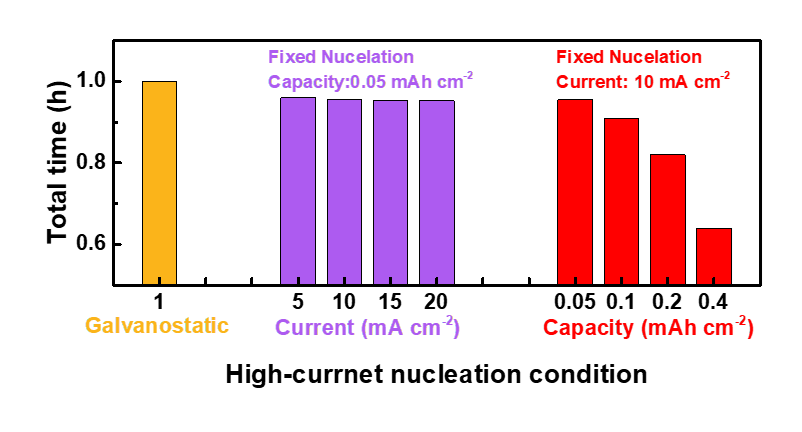


**Figure S14**. Calculated total time taken to complete the plating under galvanostatic condition and dynamic charging condition with various high-current nucleation current densities and capacities.


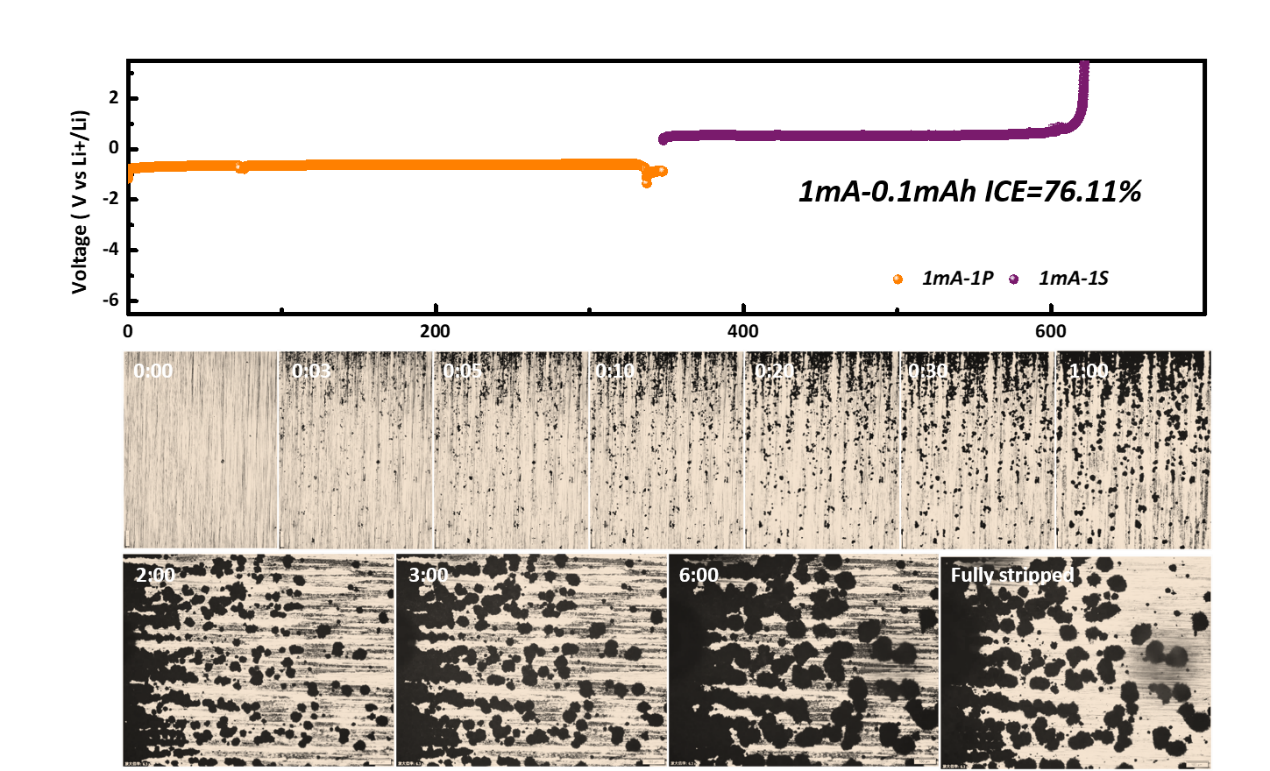


**Figure S15**. Electrochemical curves and time-dependent in-situ optical images during galvanostatic plating/stripping at 1 mA.


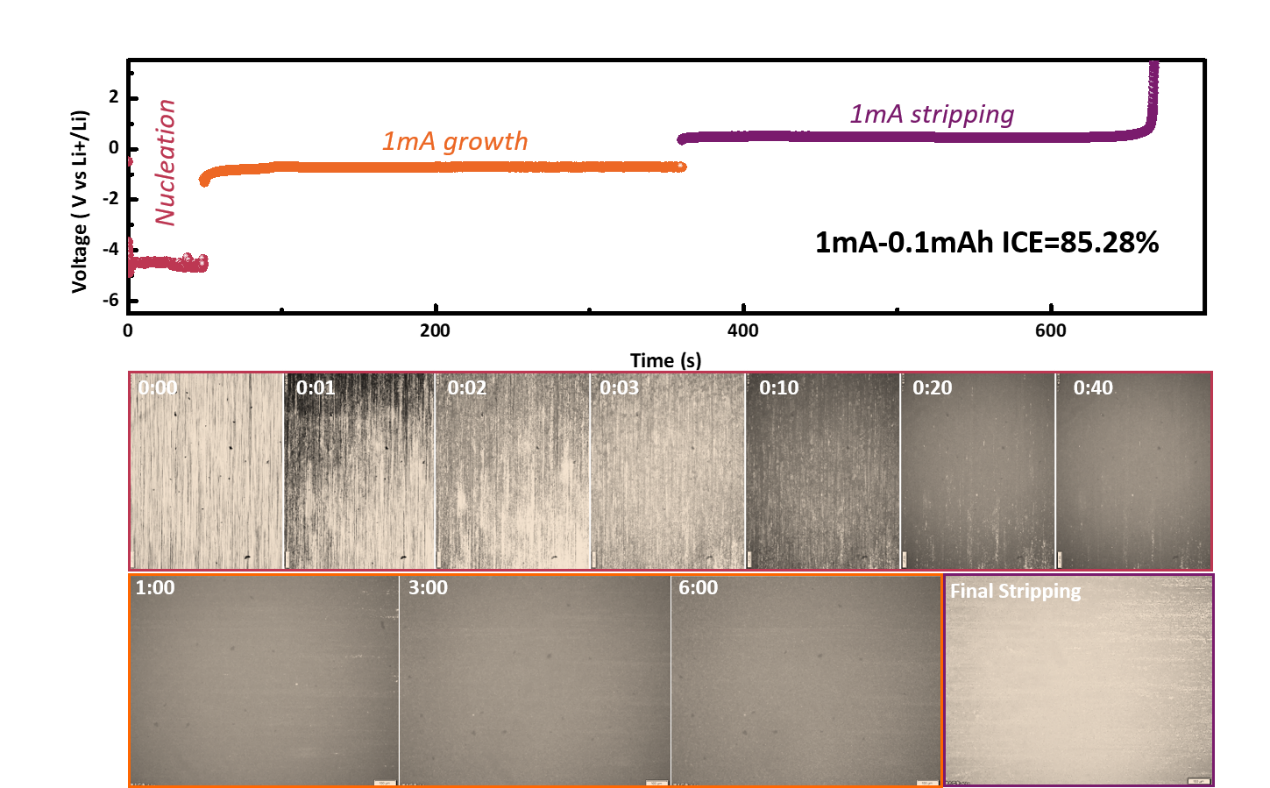


**Figure S16**. Electrochemical curves and time-dependent in-situ optical images with step-wise plating.


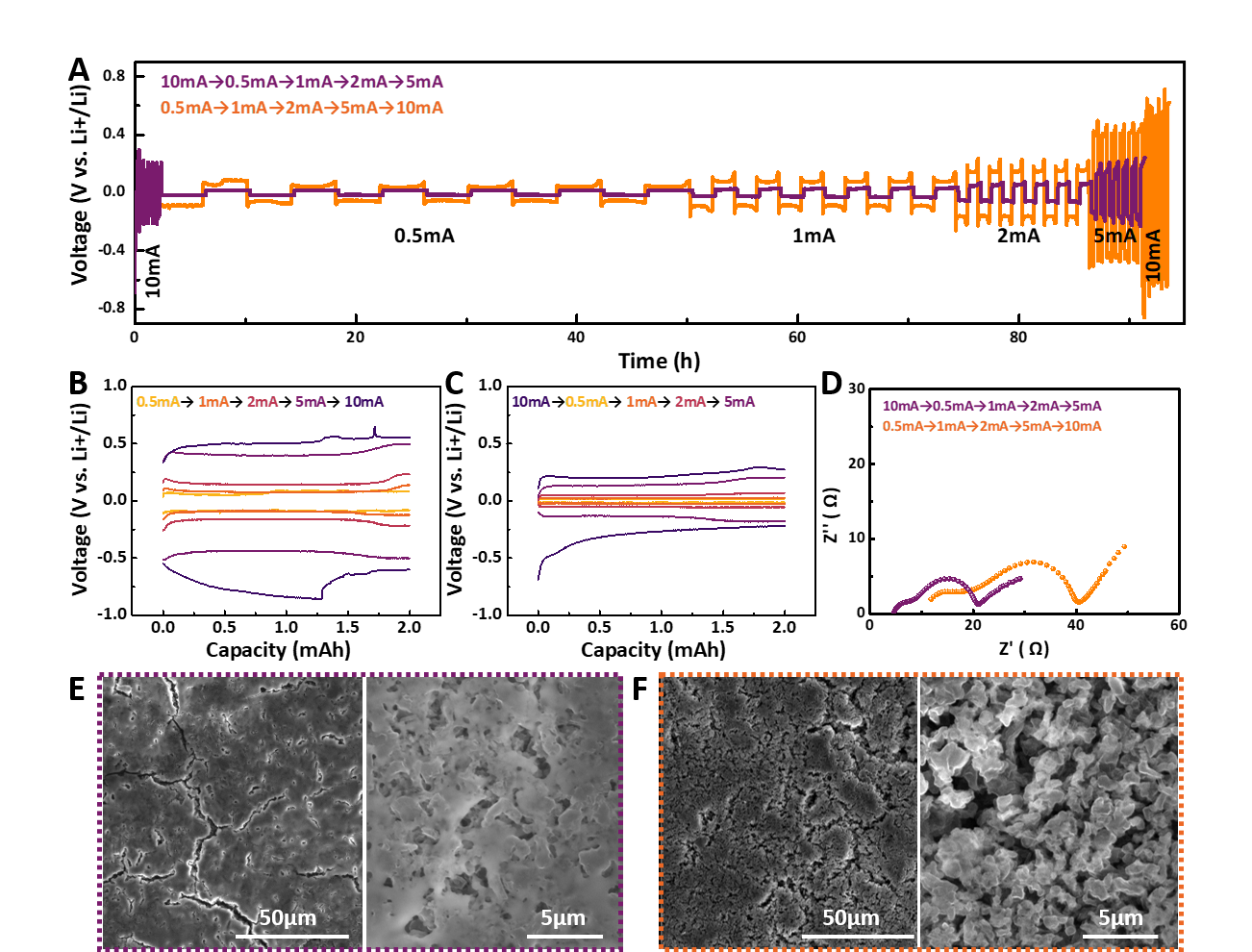


**Figure S17.** a) Rate capability of Li-Li symmetric cells starting with 0.5 or 10 mA cm^-2^. Voltage hysteresis of cells starting with b) 0.5 mA cm^-2^ and c) 10 mA cm^-2^. Electrochemical impedance spectra of cells after testing with the two different current settings. SEM image of Li anode after testing with a starting current of e) 10 mA cm^-2^ and f) 0.5 mA cm^-2^.
